# The mediating role of job satisfaction and presenteeism on the relationship between job stress and turnover intention among primary health care workers

**DOI:** 10.1186/s12939-023-01971-x

**Published:** 2023-08-15

**Authors:** Liangwen Ning, Huanhuan Jia, Shang Gao, Minghui Liu, Jiaying Xu, Sangyangji Ge, Ming Li, Xihe Yu

**Affiliations:** 1 School of Public Administration, Jilin University, Changchun City, Jilin Province China; 2 School of Public Health, Jilin University, Changchun City, Jilin Province China

**Keywords:** Primary health care workers, Turnover intention, Job stress, Job satisfaction, Presenteeism

## Abstract

**Background:**

Turnover problems among primary health care workers are a significant contributor to the shortage of health human resources. This study aims to determine the relationship between job stress and turnover intention among primary health care workers, as well as to examine the mediating effects of job satisfaction and presenteeism on this relationship.

**Methods:**

Stratified random sampling and quota sampling were used to select 703 primary health care workers in Jilin Province, China in January 2020. Validated scales were used to measure turnover intention, job stress, job satisfaction, and presenteeism among primary health care workers. The study utilized a partial least squares structural equation modeling (PLS-SEM) approach to test the research hypotheses.

**Results:**

The turnover intention score of primary health care workers in Jilin Province was 2.15 ± 1.03, and 19.5% of respondents reported a higher turnover intention. Significant sex and occupation differences were found, with a higher rate of turnover intention for male and doctor groups among primary health care workers. This study also revealed a positive correlation between job stress and turnover intention (*β* = 0.235, *P* < 0.001), a significant negative correlation between job satisfaction and turnover intention (*β*= -0.347, *P* < 0.001), and a significant positive correlation between presenteeism and turnover intention (*β* = 0.153, *P* < 0.001). Moreover, the study revealed a significant indirect effect of job stress on turnover intention which was mediated by job satisfaction (*β* = 0.183, *P* < 0.001) and presenteeism (*β* = 0.078, *P* < 0.001).

**Conclusion:**

We confirmed the positive association between job stress and presenteeism with turnover intention, as well as the negative association between job satisfaction and turnover intention. Moreover, our study confirmed the mediating role of job satisfaction and presenteeism in the relationship between job stress and turnover intention. This study provides scientific evidence to address the turnover problem among primary health care workers.

## Background

Primary health care (PHC) plays a crucial role in providing basic medical and public health services to the population, making it a key component of a country’s health care system. Proper allocation of human resources is essential to ensure the smooth functioning of the health system, increase health service accessibility, and improve health outcomes and equity [1]. The demand for PHC is expected to rise considerably due to the aging population and the increase in chronic diseases. However, the global human resource crisis for health in primary care institutions has become a pressing issue. A study predicts that the United States will face a shortage of 7,300 to 43,100 primary care physicians by 2030 [2]. In response, the Chinese government has introduced policies such as the Healthy China 2030 Plan aimed at strengthening the role of primary health care [3, 4], and has increased funding to relevant institutions more than tenfold since 2008 [5, 6]. Despite these efforts, the shortage of human resources in primary health care institutions in China remains a serious problem [7]. Turnover is a major cause of human resource shortages in primary health care, which not only significantly increases the cost of operating hospitals but may also compromise the quality of care [8]. According to the National Health Commission of the People’s Republic of China, the ratio of the number of primary health care workers to total number of health personnel in China decreased from 40.0 to 31.7% between 2010 and 2021 [9, 10].

Turnover intention is the likelihood that an employee will voluntarily leave their job in the future [11]. Numerous studies have shown that turnover intention is a reliable predictor of actual turnover behavior and reflects the management level of an organization [12]. High rates of turnover intention among health care professionals in teaching, tertiary, general, district, or acute hospitals have been observed in studies conducted in Iraq [13] and Italy [14]. Since the COVID-19 outbreak, studies have found that objective reasons such as increased workload, virus exposure, and isolation from family members have heightened the psychological stress experienced by health care workers [15], potentially contributing to separation issues [16]. A survey conducted in the United States revealed that 18% of health care workers left their jobs due to the COVID-19 pandemic [17], and health care workers in Singapore resigned due to fatigue during the COVID-19 outbreak [18]. Previous studies on turnover intention have largely focused on medical personnel in hospitals, with relatively little attention given to primary health care workers.

Numerous studies have investigated the factors that influence physicians’ willingness to leave their jobs, including external environmental factors such as the national medical system and occupational environment [19], salary [19], and doctor‒patient relationship [20], as well as internal individual factors such as sex and age [21]. In recent years, there has been an increasing focus on the influence of psychological factors, such as job satisfaction and job stress, on employees’ intention to leave [22]. Job satisfaction has been a key variable in studies related to turnover intention, and research in many countries has confirmed a significant and negative relationship between job satisfaction and the intention to leave among health care workers [23, 24]. Another key predictor of turnover intention is job stress. High levels of stress can lead to psychological and emotional exhaustion, making employees more vulnerable to leaving their jobs. Studies in many countries have shown a positive relationship between job stress and turnover intention among health care workers [22, 25].

However, there are limited research focusing on the impact of presenteeism on the intention of primary health care workers to leave their jobs. Presenteeism refers to a situation where an employee continues to work despite being sick or on vacation [26]. Once considered a positive behavior and the opposite of absenteeism, presenteeism is now viewed as negative and avoidable as it reduces both individual and organizational productivity. Presenteeism is especially prevalent among health care workers due to excessive workloads and high levels of stress [27]. A study conducted in Hong Kong revealed that presenteeism among medical staff can have serious consequences on physical health, well-being, and turnover rates [28].

Based on the above discussion, this study explored the relationship between job stress, job satisfaction presenteeism and turnover intention among primary health care workers. Given the strengths of the structural equation modeling (SEM) approach compared to logistic regression and multi-factor linear regression, particularly in assessing relationships involving multiple variables, this study utilized the partial least squares structural equation modeling (PLS-SEM) method to effectively analyze and validate the variables under investigation.

## Literature review

### Job stress and turnover intention

According to Lazarus and Folkman, stress is the result of interactions between individuals and their environment [29]. The effort-reward imbalance (ERI) model suggests that stress arises from a discrepancy between one’s high effort and commitment at work and the low rewards received in return [30]. The job demand-resources (JD-R) theory devised by Demerouti proposes that despite differences in job content across occupations, there are risk factors associated with job stress, including job demands and job resources [31].

Previous research on turnover intention among health care workers has primarily focused on the effect of job stress [32]. For example, a study conducted on 296 nurses in Iran showed that occupational stress was positively associated with nurses’ turnover intention [33]. This association has also been observed during the COVID-19 pandemic. A study revealed that nurses working in COVID-19 triage hospitals in Egypt experienced increased physical and psychological stress, leading to a higher likelihood of leaving their jobs [34].

Therefore, we postulate the following hypothesis:

*H1: Primary health care workers’ job stress will have a positive association with turnover intention.*

### Job satisfaction and turnover intention

Job satisfaction is a multidimensional concept that refers to an employee’s evaluation and attitude toward various aspects of their job content and environment [12]. Mobley’s heuristic model of the employee withdrawal decision process suggests that job satisfaction is significantly associated with turnover intention [35]. Numerous studies have found that job satisfaction is a crucial antecedent variable that influences the turnover intention of medical staff, with a negative association between job satisfaction and turnover intention [36–38]. A 2017 survey of Chinese general practitioners showed that job satisfaction had a direct negative association with their intention to leave [39]. Similarly, an Egyptian survey conducted during the COVID-19 pandemic demonstrated that the job satisfaction of frontline physicians was a significant negative predictor of their turnover intention [40].

In addition to its direct effect, job satisfaction can also act as a mediating factor in the relationship between job stress and intention to leave. Kuo’s survey found that job satisfaction significantly mediated the relationship between stress and turnover intention among nurses in Taiwan [41]. Similarly, a study conducted in the western provinces of China demonstrated a mediating effect of job satisfaction on the relationship between job stress and turnover intention among rural health care workers [42].

Based on theory and research, we hypothesized the following:

*H2*: *Primary health care workers’ job satisfaction will have a negative association with turnover intention.*

*H3: Primary healthcare workers’ job stress will have negative association with job satisfaction.*

*H4: Job satisfaction will play a mediating role in the relationship between job stress and the turnover intention of primary health care workers.*

### Presenteeism and turnover intention

Presenteeism refers to a situation in which an employee chooses to continue working despite being ill or unwell [43]. Apart from the negative impact on employees’ health, presenteeism can also lead to reduced productivity, errors in work, or reduced quality of service [44]. The conservation of resources (COR) theory posits that negative outcomes occur when individuals invest significant resources, such as time, energy, and opportunities, but receive fewer resources in return [45]. Primary health care workers who are unwell may need to devote more resources to work to maintain the quality of health services and performance. However, if the rewards received do not increase or even lead to further losses, individuals may resort to measures such as turnover to reduce resource losses.

Presenteeism is a prevalent issue among primary health care workers [46], with job stress and job satisfaction being significant predictors. According to Yang’s survey, heightened stress levels among health care workers reduce their work enthusiasm and increase the likelihood of presenteeism [26]. A study conducted in Korea revealed a positive correlation between occupational stress and presenteeism among shiftwork nurses [47]. Similarly, a survey on Korean occupational therapists indicated a strong association between stress and presenteeism, with presenteeism mediating the relationship between job stress and turnover intention [48].

Accordingly, we propose the following hypothesis:

H5: *Primary health care workers’ presenteeism will have a positive association with turnover intention.*

H6: *Primary health care workers’ job stress will have a positive association with presenteeism.*

H7: *Presenteeism will play a mediating role in the relationship between job stress and the turnover intention of primary health care workers.*

## Methods

### Study design and data collection

In this study, a cross-sectional survey was conducted in January 2020 among health care workers in primary care institutions in Jilin Province, China, using a field questionnaire. The sampling strategy involved a combination of stratified random sampling and quota sampling. First, the research units were categorized into 60 regions (counties or county-level cities/districts) based on administrative divisions of Jilin Province. From each region, two primary medical institutions were randomly selected. Second, two doctors, two nurses, and two medical technicians were randomly chosen from each primary care institution. The inclusion criteria for medical staff participating in the survey were: (1) being employed in a primary care institution in Jilin Province, China, and (2) agreeing to participate in the survey.

The survey received ethical approval from the Medical Ethics Committee of the School of Public Health, Jilin University (No. 20,191,203). Before commencing the study, researchers provided a detailed explanation of the survey’s objective and obtained informed consent from all participants. A total of 720 questionnaires were distributed, and 703 valid questionnaires were collected, yielding an effective response rate of 97.6%.

### Construct measurements

#### Turnover intention

Turnover intention was primarily evaluated using the Mobley Model of Employee Turnover Behavior [35]. The scale consisted of three items in this study: “I have considered quitting my current position.” “Within the next year, I plan to search for a new job.” and “If presented with the opportunity, I would definitely accept a new and improved job.” Each item was rated on a 5-point Likert scale, with higher scores indicating a stronger inclination to leave the job. In current study, the scale had good internal consistency reliability, with a Cronbach α of 0.79 [49].

#### Job stress

Job stress was assessed using the Challenge and Hindrance-related Self-reported Stress scale (C-HSS), developed by Cavanaugh [50]. The scale consists of 11 items and measures job stress as either challenge stress or hindrance stress. Challenge stress, which is related to challenging job requirements, is assessed by 6 items, while hindrance stress, which is related to the work environment, is assessed by 5 items. Each item is evaluated on a 5-point Likert scale ranging from 1 (no stress) to 5 (great stress). The C-HSS has been translated into Chinese and has demonstrated good measurement properties in previous research with Cronbach’s coefficients of 0.86 for challenge stressors and 0.76 for hindrance stressors [51].

#### Job satisfaction

The Job Satisfaction Scale used in this study was based on the Job Description Index Scale [52] and its related scale [53].The scale demonstrated an internal consistency with a Cronbach’s Alpha coefficient of 0.659. It measures health care professionals’ satisfaction with various aspects of their job, including compensation, colleague relationships, work environment, career development, hospital management, job meaning, occupational risk, workload, and overall satisfaction with nine items. A 5-point Likert scale ranging from 1 (very dissatisfied) to 5 (very satisfied) was used to rate each item, with higher scores indicating greater job satisfaction among the study participants.

#### Presenteeism

The Stanford Presenteeism Scale-6 (SPS-6), developed by Koopman et al., was used in this study to measure attendance problems among primary health care workers [54]. The Chinese version of the scale comprises two dimensions: completing work (4 items) and avoiding distractions (2 items), which measure work outcomes and processes, respectively [55]. Participants rated each item on a 5-point Likert scale ranging from 1 (strongly disagree) to 5 (strongly agree). Two items in the avoiding distraction dimension were reverse scored, while the remaining items were positively scored. The total score was calculated by summing the individual item scores, with a range of 6–30. Higher scores on the SPS-6 scale indicate more severe presenteeism problems, according to Koopman et al [54]. The scale has demonstrated good internal consistency reliability in the Chinese population, as indicated by a Cronbach’s coefficient of 0.692 [55].

In addition, we also collected demographic characteristics of the survey respondents, including their sex, age, marital status, education level, professional title, department, and years of work experience.

### Data analysis

We performed one-way ANOVA and t-tests using SPSS software (IBM SPSS Statistics 25; SPSS, Inc., Chicago, IL, USA) to examine the differences in turnover intention, job stress, job satisfaction, and presenteeism of the population by demographic characteristics. One-way ANOVA was used for comparisons among multiple groups, and t test was used for comparisons between two groups. In addition, a partial least squares structural equation modeling (PLS-SEM) analysis was performed using Smart PLS 3.2.9 (SmartPLS GmbH, Bönningstedt, Germany) in this study. Compared to covariance-based structural equation modeling (CB-SEM), PLS-SEM exhibits greater advantages in exploratory research for theory development, as such studies typically involve complex and unclear relationships among variables [56]. PLS-SEM analysis consists of two main steps. The first step involves assessing the reliability and validity of each construct in the measurement model, while the second step involves estimating the structural model and examining each hypothesis.

In this study, the reliability and validity of the measurement model were evaluated using indicator loadings, internal consistency reliability, convergent validity, and discriminant validity as criteria. According to Hair, indicator loadings above 0.708 are recommended [57]. To assess the internal consistency of the constructs, Cronbach’s alpha coefficient (α), composite reliability (CR) and rho_A were utilized. As per Hair et al., Cronbach’s alpha, CR and rho_A values above 0.7 are considered good, and values between 0.6 and 0.7 are considered acceptable levels of internal consistency reliability [56]. The convergent validity of the measurement model was assessed by the average variance extracted (AVE). An AVE value of above 0.50 was considered adequate. The discriminant validity of the model was assessed using the Fornell-Larcker criterion and heterotrait- monotrait (HTMT) ratio of the correlations. The Fornell-Larcker criterion for examining the discriminant validity of latent variables is whether the AVE of a construct is greater than the square of the correlation coefficient between it and other constructs. An HTMT value less than 0.90 indicates good discriminant validity [58].

## Results

### Demographic characteristics

As depicted in Table 1, 73.8% of the participants were female, while 26.2% were male. Of the total respondents, 20.2% were primary health care workers aged between 18 and 30 years, 50.5% were aged between 31 and 45 years, and 29.3% were aged 45 years or older. The majority of the participants (83.1%) were married, while 16.9% were unmarried, divorced, or widowed. Among the respondents, 63.9% had less than a junior college education, and 36.1% had a bachelor’s degree or higher. Approximately half of the participants had worked less than 10 years, 21.9% had worked for 10–20 years, and 29.0% had worked for more than 20 years.

**Table 1:** Demographic characteristics and turnover intention of respondents

Characteristics	*n* (%)	Turnover intention scores			Turnover intention scores > 3		
		M ± SD	*t/F*	*P*	*n* (%)	*χ2*	*P*
**Sex**			3.230	0.002		9.384	0.002
Male	184(26.2)	2.36 ± 1.10			50(27.2)		
Female	519(73.8)	2.07 ± 0.99			87(16.8)		
**Age**			1.091	0.337		2.062	0.357
18–30	142(20.2)	2.10 ± 1.06			26(18.3)		
31–45	355(50.5)	2.12 ± 1.01			64(18.0)		
> 45	206(29.3)	2.24 ± 1.03			47(22.8)		
**Marital status**			-0.287	0.774		0.656	0.418
Unmarried/Divorced/widowed	119(16.9)	2.12 ± 1.03			20(16.8)		
Married	584(83.1)	2.15 ± 1.03			117(20.0)		
**Education level**			-1.351	0.177		6.139	0.013
Junior college or below	449(63.9)	2.11 ± 1.00			75(16.7)		
Bachelor’s degree or above	254(36.1)	2.22 ± 1.08			62(24.4)		
**Occupation**			4.774	0.009		9.039	0.011
Doctor	277(39.4)	2.29 ± 1.05			69(24.9)		
Nurse	243(34.6)	2.03 ± 0.98			36(14.8)		
Technician	183(26.0)	2.09 ± 1.04			32(17.5)		
**Working years**			1.132	0.323		1.915	0.384
< 10	345(49.1)	2.16 ± 1.01			64(18.6)		
10–20	154(21.9)	2.23 ± 1.05			36(23.4)		
> 20	204(29.0)	2.07 ± 1.03			37(18.1)		
**Total**	703(100.0)	2.15 ± 1.03			137(19.5)		

### Results of turnover intention

Regarding the participants’ turnover intention, 19.5% of the respondents gave a rating higher than 3 out of a possible score of 5. The mean score of turnover intention for all subjects was 2.15 with a standard deviation of 1.03. The respondents who were male, had a bachelor’s degree or above, and were doctors had a higher turnover intention. The turnover intention scores of primary health care workers with different characteristics are demonstrated in Table 1.

### Results of job stress, job satisfaction and presenteeism

The scores of job stress, job satisfaction, and presenteeism are shown in Table 2. The score of job stress was 22.49 ± 8.65, 32.36 ± 7.16 for job satisfaction, and 13.03 ± 4.43 for presenteeism. The results of the ANOVA indicate significant differences in the job stress scores among the groups based on sex (*P* < 0.001), age (*P* < 0.001), occupation (*P* < 0.001), and years of work experience (*P* = 0.023). Similarly, significant differences were noted in the job satisfaction scores among the groups based on sex (*P* = 0.001), age (*P* < 0.001), marital status (*P* = 0.014), occupation (*P* = 0.007), and years of work experience (*P* = 0.001). Furthermore, there was a significant difference in the presenteeism scores based on sex (*P* < 0.001) according to the analysis of variance.

**Table 2:** Results of job stress, job satisfaction and presenteeism

Characteristics	Job Stress			Job Satisfaction			Presenteeism		
	Mean ± SD	*t/F*	*P*	Mean ± SD	*t/F*	p	Mean ± SD	*t/F*	*P*
**Sex**		3.998	<0.001		-3.192	0.001		4.277	<0.001
Male	24.66 ± 8.99			30.92 ± 6.97			14.21 ± 4.49		
Female	21.72 ± 8.40			32.87 ± 7.17			12.61 ± 4.33		
**Age**		8.317	<0.001		8.655	<0.001		1.779	0.170
18–30	20.16 ± 8.11			34.33 ± 7.40			12.54 ± 4.27		
31–45	22.55 ± 8.52			32.30 ± 6.98			12.98 ± 4.49		
> 45	23.98 ± 8.92			31.12 ± 7.05			13.44 ± 4.41		
**Marital status**		-1.645	0.100		2.464	0.014		0.334	0.738
Unmarried/Divorced/widowed	21.30 ± 8.18			33.83 ± 6.62			13.14 ± 4.07		
Married	22.73 ± 8.73			32.06 ± 7.24			13.00 ± 4.50		
**Education level**		-1.683	0.093		0.144	0.886		1.206	0.228
Junior college or below	22.08 ± 8.64			32.39 ± 7.13			13.18 ± 4.26		
Bachelor’s degree or above	23.22 ± 8.64			32.31 ± 7.23			12.75 ± 4.70		
**Occupation**		9.997	<0.001		4.966	0.007		2.931	0.054
Doctor	24.27 ± 8.86			31.36 ± 7.04			13.41 ± 4.45		
Nurse	21.49 ± 8.66			33.30 ± 7.04			12.49 ± 4.41		
Technician	21.12 ± 7.85			32.62 ± 7.35			13.16 ± 4.37		
**Working years**		3.802	0.023		7.416	0.001		1.185	0.306
< 10	21.58 ± 8.36			33.40 ± 7.20			12.77 ± 4.34		
10–20	23.53 ± 8.56			31.08 ± 6.96			13.38 ± 4.26		
> 20	23.24 ± 9.08			31.58 ± 7.02			13.19 ± 4.69		
**Total**	22.49 ± 8.65			32.36 ± 7.16			13.03 ± 4.43		

### Measurement model assessment

The reliability and validity results of the latent construct are presented in Table 3. The indicator loadings for each item of job stress, job satisfaction, presenteeism, and turnover intention were in the range of 0.718–0.890, 0.683–0.859, 0.870–0.964, and 0.849–0.909, respectively. One item had an indicator loading slightly below the standard value of 0.708 (the indicator loading for JS2 was 0.683). After comprehensive consideration, we decided to retain the item.

Cronbach’s α coefficients for job stress, job satisfaction, presenteeism, and turnover intention were 0.926, 0.930, 0.815 and 0.853, while the rho_A values were 0.930, 0.937, 0.906 and 0.862, and the CR values were 0.938, 0.942, 0.868 and 0.911, respectively. All values of Cronbach’s α coefficients, the rho_A and CR for each latent variable were greater than 0.7, indicating good internal consistency reliability for each variable.

The AVE values for the constructs of job stress, job satisfaction, presenteeism, and turnover intention were 0.582, 0.643, 0.560 and 0.773, respectively. These AVE values were all above 0.5, indicating good convergent validity for each latent variable.

**Table 3:** Reliability and validity analysis

Variables	Items	Indicator Loading	Cronbach’sα	rho_A	CR	AVE
**Job Stress**			0.926	0.930	0.938	0.582
Challenge Stress	CS1	0.861	0.936	0.936	0.949	0.758
	CS2	0.878				
	CS3	0.890				
	CS4	0.881				
	CS5	0.860				
	CS6	0.853				
Hindrance Stress	HS1	0.718	0.846	0.854	0.890	0.619
	HS2	0.773				
	HS3	0.812				
	HS4	0.842				
	HS5	0.784				
**Job Satisfaction**	JS1	0.759	0.930	0.937	0.942	0.643
	JS2	0.683				
	JS3	0.791				
	JS4	0.859				
	JS5	0.813				
	JS6	0.837				
	JS7	0.794				
	JS8	0.819				
	JS9	0.847				
**Presenteeism**			0.815	0.906	0.868	0.560
Completing Work	CW1	0.908	0.927	0.928	0.948	0.820
	CW2	0.929				
	CW3	0.915				
	CW4	0.870				
Avoiding Distraction	AD1	0.964	0.886	0.964	0.945	0.895
	AD2	0.928				
**Turnover Intention**	TI1	0.909	0.853	0.862	0.911	0.773
	TI2	0.849				
	TI3	0.878				

The findings presented in Table 4 demonstrate that the square root value of the AVE value for each construct was larger than the correlation coefficient of all the other constructs in the same row and column. This indicates that the structure possessed sufficient discriminant validity. In addition, the result of 0.496 indicated a significant positive association between job stress and turnover intention. The significant inverse relationship between job satisfaction and turnover intention was confirmed by the value of -0.528. Moreover, a significant positive association between presenteeism and turnover intention was demonstrated by the value of 0.401.

**Table 4:** Fornell-Larcker criterion

	Job Stress	Job Satisfaction	Presenteeism	Turnover Intention
**Job Stress**	0.763			
**Job Satisfaction**	-0.527	0.802		
**Presenteeism**	0.510	-0.370	0.749	
**Turnover Intention**	0.496	-0.528	0.401	0.879

Table 5 presents the results of the heterotrait-monotrait (HTMT) ratio metrics, which demonstrate good discriminant validity among the latent variables of job stress, job satisfaction, presenteeism, and turnover intention. All HTMT values ranged from 0.428 to 0.584, which is below the cutoff value of 0.90.

**Table 5:** Heterotrait-monotrait (HTMT)

	Job Stress	Job Satisfaction	Presenteeism	Turnover Intention
Job Stress				
**Job Satisfaction**	0.555			
**Presenteeism**	0.541	0.428		
**Turnover Intention**	0.555	0.584	0.437	

### Assessment of the structural model

Table 6 presents the results of the structural model. The findings indicated that job stress was positively correlated with turnover intention (*β* = 0.235, *P* < 0.001), while job satisfaction was negatively correlated with turnover intention (*β*=-0.347, *P* < 0.001) and presenteeism was positively correlated with turnover intention (*β* = 0.153, *P* < 0.001). These results support H1, H2, and H5.

Furthermore, the results revealed a significant relationship between job stress and both job satisfaction (*β*=-0.527, *P* < 0.001) and presenteeism (*β* = 0.510, *P* < 0.001), providing support for H3 and H6.

**Fig. 1:**
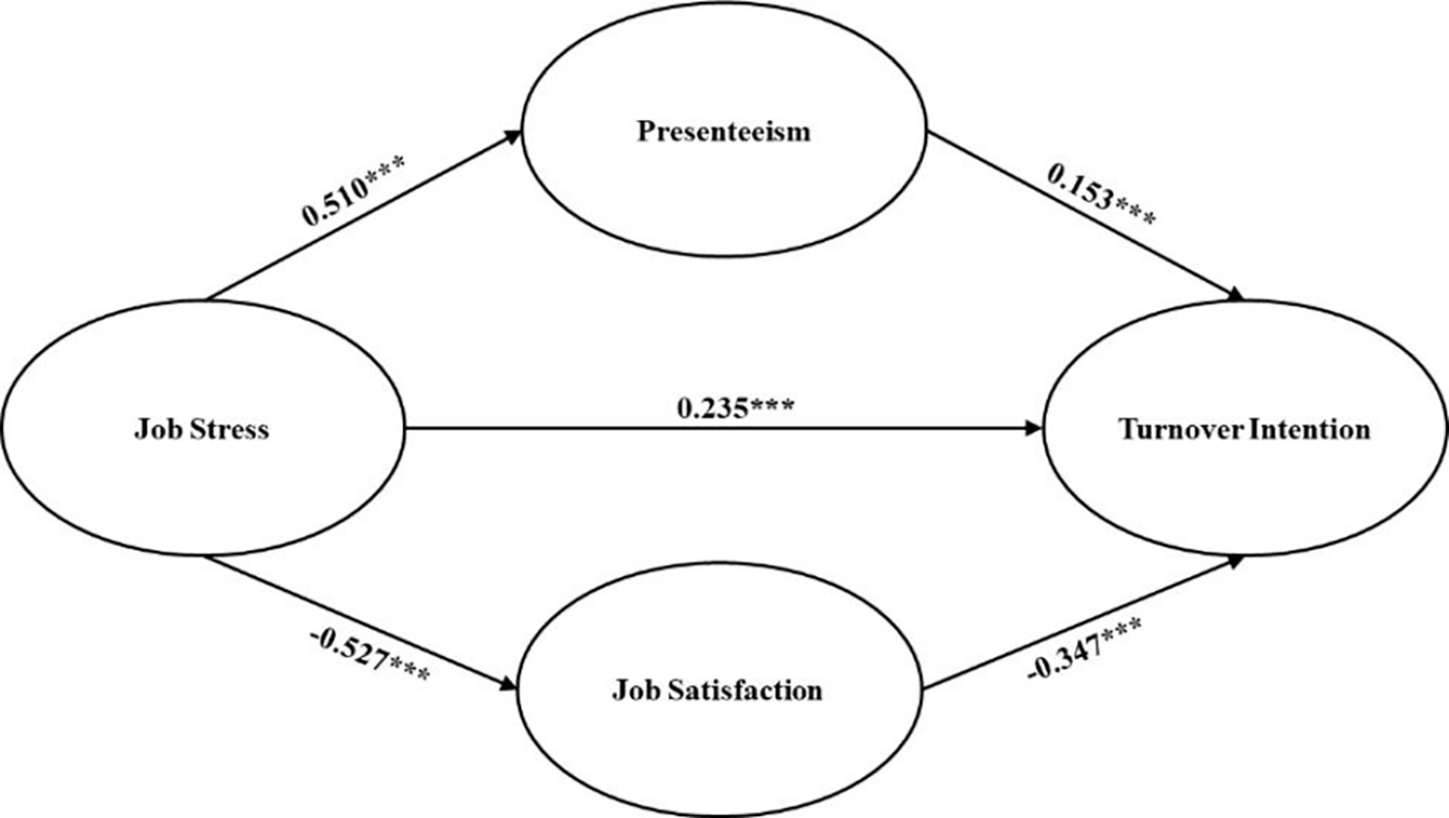
Structural equation model Note: ^***^p<0.001

In addition, the study found a significant indirect effect of job stress on turnover intention through the mediating roles of job satisfaction (*β* = 0.183, *P* < 0.001) and presenteeism (*β* = 0.078, *P* < 0.001). These results support our hypothesized models (H4, H7). The diagram of the proposed model is illustrated in Fig. 1.

**Table 6:** Hypothesis results

Hypothesis	Path	Path Coefficient	*P*	95% BCa Confidence Interval
H1	Job Stress‒>Turnover Intention	0.235	< 0.001	0.152 ~ 0.321
H2	Job Satisfaction‒>Turnover Intention	-0.347	< 0.001	-0.421 ~ -0.267
H3	Job Stress‒>Job Satisfaction	-0.527	< 0.001	-0.583 ~ -0.464
H4	Job Stress‒>Job Satisfaction‒>Turnover Intention	0.183	< 0.001	0.139 ~ 0.231
H5	Presenteeism‒>Turnover Intention	0.153	< 0.001	0.072 ~ 0.234
H6	Job Stress‒>Presenteeism	0.510	< 0.001	0.432 ~ 0.577
H7	Job Stress‒>Presenteeism‒>Turnover Intention	0.078	< 0.001	0.036 ~ 0.123

## Discussion

Turnover intention among primary health care workers is a pressing issue that requires prompt attention. Adequate resource allocation for primary health care workers is crucial for ensuring universal and equitable high-quality health care [4]. The aim of this study was to examine the associations of job stress, job satisfaction, and presenteeism with turnover intention, and to emphasize the mediating role of job satisfaction and presenteeism in the relationship between job stress and turnover among primary health care workers in China.

In this study, the mean turnover intention score among medical staff in primary health care institutions in Jilin Province, China was 2.15 (with a standard deviation of 1.03), and 19.5% of respondents reported high turnover intention. There are differences between our study’s findings and those of other countries. A study conducted in England revealed that 11.8% of primary health care doctors expressed a high intention to leave their positions [59]. In South Africa, a study demonstrated that half of the rural nurses surveyed considered turnover [60]. A meta-analysis was conducted to determine the prevalence of turnover intention among primary health care workers in China [61]. The pooled prevalence was found to be 30.4%, with Gu’s study in Shanghai City reporting the highest prevalence of 54.3% [62], and Liu’s study in Shaanxi Province reporting the lowest prevalence of 8.0% [63]. The distribution of primary care workers in China is known to be uneven [7]. The diversity in number, gender, age, and educational background of primary care staff across different regions in China may have implications for the varying levels of turnover intention observed in these areas. A national survey conducted in China has revealed that highly educated primary healthcare professionals exhibit a greater intention to leave their positions [64]. Previous survey found that the percentage of primary healthcare professionals with a college degree or higher in Jilin Province is 44.00%, lower than the national average of 55.80% [65]. This suggests that the research sample consisted of individuals with relatively lower educational attainment, which might contribute to a lower turnover intention. Furthermore, it is important to consider that the results may vary depending on the research time, and sample size [61].

In this study, the PLS-SEM analysis revealed that job stress had a positive association with turnover intention among primary health care workers. Job stress is a prevalent issue among medical staff, and previous research has demonstrated that job stress is positively associated with the turnover intention of health care workers [66]. A study of workers in primary health care centers in Saudi Arabia supported these findings [25]. With the implementation of the Health China Strategy, primary medical institutions have been assigned a more prominent role as the foundation of China’s three-tier medical and health service network. However, primary care institutions face resource constraints compared to secondary and tertiary hospitals, which presents a challenge in managing the ever-increasing workload. The heavy workload of primary care providers exposes them to a multitude of responsibilities, leading to blurred roles and high job expectations, which in turn increases their work pressure. Job stress not only increases the physiological burden of employees, but also leads to psychological burnout, resulting in work withdrawal behavior.

Our study found that job satisfaction had a negative association with turnover intention among primary health care workers. Previous research has consistently shown that job satisfaction is a crucial factor influencing turnover intention among primary care workers in China, and our results support this finding. For instance, Gu et al. found that primary health care workers in rural areas of China were dissatisfied with their work situation [67], while a study of 1,148 primary care providers in China confirmed the negative association of job satisfaction with turnover intention [68]. Similar studies conducted in various countries have shown that primary care physicians generally have low job satisfaction. A survey conducted in Malaysia, for example, found that only 62.9% of primary care providers believed that their remuneration and efforts were aligned [69].

Our study revealed a positive correlation between presenteeism and turnover intention among primary health care workers. Previous research also demonstrated that presenteeism had a significant impact on turnover intention. The demanding workload of primary care workers can lead to increased sickness behavior, which can worsen their health condition. Consequently, primary care workers with poor health may need to invest more resources to ensure the quality of their work, without receiving proportional compensation, resulting in a deteriorating work experience and ultimately a higher likelihood of leaving. Kang’s study on nurses produced similar results, with presenteeism significantly increasing the risk of departure among clinical nurses with more than six years of experience [27].

In addition, our study provided evidence supporting the mediating effect of job satisfaction on the relationship between job stress and turnover intention, which is consistent with previous research. Job satisfaction can alleviate the stress experienced by primary care workers at work, satisfy their psychological and emotional needs, and reduce their intention to leave. Liu’s study found that job satisfaction weakened the positive association between work stress and turnover intention among rural health workers by serving as a complete mediator, with reward satisfaction being the strongest mediator [42]. Similarly, Kuo’s study of long-term care workers in Taiwan confirmed the mediating effect of job satisfaction on the relationship between job stress and turnover intention [41].

This study demonstrated that presenteeism mediate the relationship between job stress and turnover intention among primary health care workers. According to the conservation of resources theory, job stress compels primary medical staff to persevere in their work even when they are ill. However, in the absence of rewards, individuals may opt to quit to minimize resource depletion. Our findings suggest that job stress may prompt workers to continue working despite feeling unwell, which can lead to disengagement and reduced commitment to the primary health care institution, ultimately resulting in increased turnover intention [29]. These results are consistent with a study by Chun, which found that presenteeism partially mediated the relationship between job stress and turnover intention among occupational therapists in Korea [48].

Our study also found that male primary health care workers and those in the doctor group had a higher rate of turnover intention. This finding is consistent with previous research conducted in China, which showed that male general practitioners were more likely to leave their jobs than their female counterparts [64]. However, Bardoel’s study found that female general practitioners were more likely to make unplanned turnover than males [70]. This could be related to the region of the research, and further research about the sex difference in primary health care workers’ turnover intention is needed to explore this issue in greater depth. In China, many studies have shown that doctors are more likely to be dissatisfied with their jobs due to workload and the doctor‒patient relationship [71], which could explain why the turnover intention of doctors tends to be higher, as observed in our study.

Our research has some limitations that need to be considered. First, the study population only included primary health care workers in Jilin Province, which may limit the generalizability of the findings to other regions of China. Second, the cross-sectional design of the study does not allow us to draw any causal inferences.

## Conclusions

Our study investigated the relationship between turnover intention and job stress, job satisfaction, and presenteeism among health care professionals working in primary health care institutions in Jilin Province, China. We found that job stress and presenteeism had a positive association with turnover intention, while job satisfaction was negatively associated with turnover intention. Furthermore, our study confirmed the mediating role of job satisfaction and presenteeism in the relationship between job stress and turnover intention. These findings provide scientific evidence regarding the problem of turnover among primary health care workers and highlight the necessity of reducing job stress to improve job satisfaction and presenteeism. Therefore, it is crucial for managers to develop and implement effective measures to mitigate job stress among primary health care workers.

## Data Availability

The datasets supporting the conclusions are available from the corresponding author (Yu X, xhyu@jlu.edu.cn) on reasonable request.

## List of abbreviations

PHC: Primary health care
PLS-SEM: Partial least squares structural equation modeling
CB-SEM: Covariance-based structural equation modeling
CR: Composite reliability
AVE: Average variance extracted
HTMT: Heterotrait-monotrait
CS: Challenge stress
HS: Hindrance stress
JS: Job satisfaction
CW: Completing work
AD: Avoiding distraction
TI: Turnover intention
